# Photoluminescence properties of a Ce^3+^ doped Sr_3_MgSi_2_O_8_ phosphor with good thermal stability

**DOI:** 10.1039/c8ra00526e

**Published:** 2018-04-25

**Authors:** Baochen Wang, Yangai Liu, Zhaohui Huang, Minghao Fang

**Affiliations:** Beijing Key Laboratory of Materials Utilization of Nonmetallic Minerals and Solid Wastes, National Laboratory of Mineral Materials, School of Materials Science and Technology, China University of Geosciences Beijing 100083 China liuyang@cugb.edu.cn +86-10-82322186 +86-10-82322186

## Abstract

This study mainly reports an investigation about the crystal structure and photoluminescence properties of a Ce^3+^ doped alkaline earth metal silicate Sr_3_MgSi_2_O_8_. X-ray powder diffraction (XRD) and structure refinement methods are adopted to characterize the phase composition and crystal structure. The excitation/emission spectra and diffuse reflection spectra (DRS) of the title phosphor are measured and the mechanism of concentration quenching as well as thermal quenching are discussed in detail. Results show that the concentration quenching in Sr_3_MgSi_2_O_8_ is due to dipole–dipole interaction energy transfer between Ce^3+^ ions. The title phosphor has an excellent thermal stability with 90% emission intensity reserve when the temperature rises to 150 °C. The related mechanism is considered to be the thermal ionization effect and an energy level diagram was proposed to show the thermal ionization process.

## Abstract 2

This study mainly reports an investigation about the crystal structure and photoluminescence properties of a Ce^3+^ doped alkaline earth metal silicate Sr_3_MgSi_2_O_8_.

## Graphical abstract

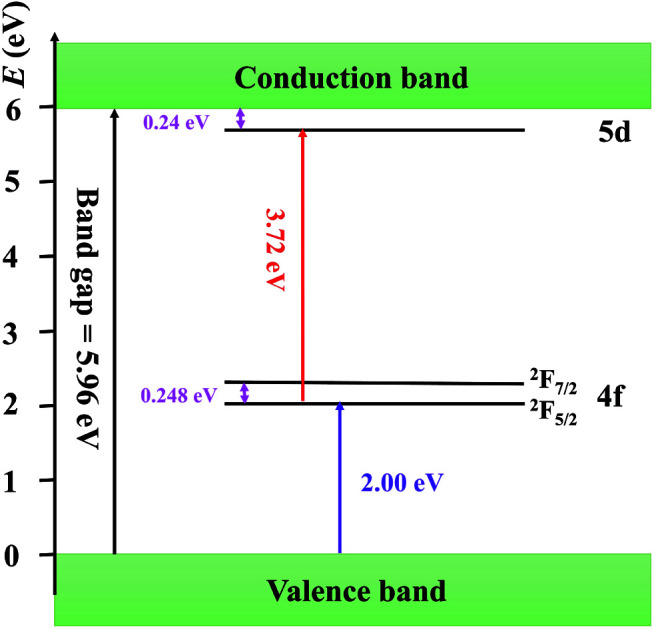

## 1. Introduction

In recent decades, white light emitting diodes (w-LEDs) have been replacing conventional incandescent and fluorescent lamps for general illumination due to their overwhelming merits such as long life, being energy saving, and their environmental friendliness as well as high efficiency.^1–3^ Although LEDs have been applied in many fields, the luminescence properties of w-LEDs for general illumination still need to be improved in terms of their color rendering index (CRI) and correlated color temperature (CCT).^4,5^ The first and most common w-LED, which is fabricated with a blue LED chip and yellow-emitting YAG:Ce^3+^ phosphor, suffers from many drawbacks such as poor CRI (*R*_a_ = 70 to 80) and high CCT (approximately 7750 K).^3,6^ To achieve “warmer” white light with high CRI, a strategy combining a blue-LED and yellow-emitting phosphors with a broader spectra covering the red/green region, or UV-LED (380 to 420 nm) and red, green, and blue (RGB) multi-compositional phosphors are proposed.^2,7,8^ Considering these approaches, developing highly efficient phosphors with high thermal quenching temperatures and tunable color points in the entire visible region under blue or UV light excitation is urgently needed.

The alkali-earth silicate is a significant host for rare earth doped phosphors due to its inherent advantages such as excellent chemical and thermal stability as well as the low price of high-purity silicate.^9^ Moreover, silicate can be produced at lower temperatures than nitrides and aluminates.^10,11^ M_3_MgSi_2_O_8_ (M = Ba, Sr, Ca) compounds have attracted much attention as promising host materials for Eu^2+^-doped blue phosphors. The emission properties have been investigated for improving the intensity and chromaticity.^12–14^ The X-ray powder diffraction (XRD) data of Sr_3_MgSi_2_O_8_ were first provided by Klasens *et al.* in 1957.^11^ After that, G. Blasse *et al.*^15^ systematically investigated the photoluminescent performance of Eu^2+^ doped Me_3_MgSi_2_O_8_ (Me = Ca, Sr, Ba) ternary system and revealed a systematic emission-color shift from blue to green depending on Me^2+^ ion size. These results are indicative of its great potential as a blue phosphor replacing commercial BaMgA_11_O_17_:Eu^2+^ (BAM). In 2009, Yoshinori Yonesaki *et al.*^16^ firstly report the precise crystal structure of Sr_3_MgSi_2_O_8._ It shows that Sr_3_MgSi_2_O_8_ crystallizes in a monoclinic system, with the cell parameters *a* = 13.877 Å, *b* = 5.458 Å, *c* = 9.452 Å. Although several reports on the photoluminescence properties of Ce^3+^/Tb^3+^ or Ce^3+^/Dy^3+^ co-doped Sr_3_MgSi_2_O_8_ phosphors have been published,^17–19^ systematic investigation on Ce^3+^ single doped Sr_3_MgSi_2_O_8_ is still very necessary. Our study is distinguished from the previous study because of systematic investigation on the phase, crystal structure and luminescent properties of title phosphor, with some new preparation conditions and new luminescent performance accordingly.

In this paper, we report the synthesis, crystal structure and photoluminescence properties of Ce^3+^ doped Sr_3_MgSi_2_O_8_ phosphor. The phase composition was characterized by XRD and the crystal structure is refined by structure refinement. The DRS, excitation and emission spectrum of title phosphor were measured and the mechanisms of concentration quenching and thermal quenching were discussed.

## 2. Experimental details

### 2.1 Raw materials and synthesis

The Sr_3_MgSi_2_O_8_:*x*Ce^3+^ phosphors were synthesized by traditional high-temperature solid-state reaction method. The starting materials are MgO (AR, Westlong Share Ltd., Guangdong, China), SrCO_3_ (99.9%, Aladdin Share Ltd., Shanghai, China), SiO_2_ (AR, Sinopharm Group Chemical Reagent Ltd., Shanghai, China) and CeO_2_ (4 N, Minmetals Rare Earth Ltd., Beijing, China). A 5 wt% extra amount of H_3_BO_3_ (99.9%, Aladdin Share Ltd., Shanghai, China) was added as a flux to promote the crystallization of title phosphors. First, certain amounts of the starting materials were thoroughly grounded in an agate mortar. Then, the mixtures were placed in alumina crucibles and sintered at 1450 °C for 4 h in a flowing reducing (10 vol% H_2_/90 vol% N_2_) atmosphere. Finally, the samples were furnace-cooled to room temperature and grounded into powders for further measurements.

### 2.2 Measurement and characterization

The phase of the title phosphor was identified by X-ray powder diffraction (XRD; D8 FOCUS diffractometer, Germany) with graphite-monochromatized Cu Kα radiation (*λ* = 1.5406 Å). The photoluminescence emission (PL) and photoluminescence excitation (PLE) spectra were measured by F-4600 fluorescence spectrophotometer (Hitachi, Japan) with a photomultiplier tube functioning at 500 V and a 150 W Xe lamp as the excitation source. The spectral resolution for photoluminescence measurement was 0.2 nm. The temperature dependent luminescence properties were determined on the same spectrophotometer equipped with an automatic temperature-regulating device. Diffuse reflection spectra were obtained *via* a UV-3600 UV-Vis-NIR spectrophotometer (Shimadzu) connected with an integrating sphere.

## 3. Result and discussion

### 3.1 Phase and crystal structure

**Fig. 1:**
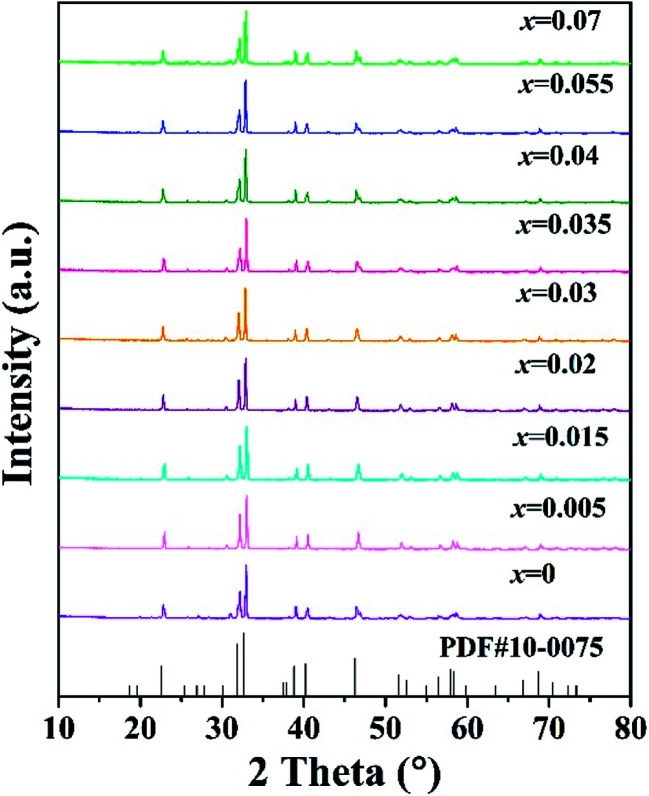
XRD patterns of the as-prepared Sr_3_MgSi_2_O_8_:*x*Ce^3+^ (*x* = 0.005, 0.015, 0.02, 0.03, 0.035, 0.04, 0.055 and 0.07) phosphors, together with the standard ICDD cards of Sr_3_MgSi_2_O_8_.

The phase composition of all the samples was characterized by XRD and the XRD patterns are shown in Fig. 1. The standard ICDD cards of Sr_3_MgSi_2_O_8_ (PDF no. 10-0075) is also illustrated in Fig. 1 as a comparison. The diffraction peaks of the as-prepared samples were consistent with those in the standard ICDD card, suggesting that all samples were obtained as pure-phase Sr_3_MgSi_2_O_8_ phase. The doping by small amounts of Ce^3+^ ions did not destroy the host crystal structure or produce foreign impurities.

**Fig. 2:**
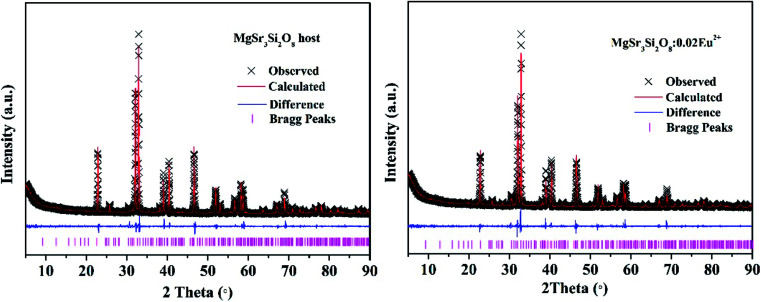
XRD refinement patterns of Sr_3_MgSi_2_O_8_ host (left) and Sr_3_MgSi_2_O_8_:0.02Ce^3+^ (right) phosphor. Black crosses represent the observed diffraction peak; red lines indicate the refined patterns; blue lines are residuals and purple tick marks show the Bragg positions.

To further determine the phase purity and crystal structure, the XRD patterns of Sr_3_MgSi_2_O_8_ host and Sr_3_MgSi_2_O_8_:0.02Ce^3+^ phosphor were refined by the Rietveld refinement method using the Topas program. The standard structure of Sr_3_MgSi_2_O_8_ is referenced as an initial structural model.^16^ The refinement patterns are illustrated in Fig. 2 and the main refinement parameters and detailed crystallographic data are given in Table 1. All structure refinements are convergent and end with acceptable and publishable *R* factors. The results of the refinement further demonstrate that these phosphors match well with the starting model (Sr_3_MgSi_2_O_8_) and doping of Ce^3+^ ions don't bring any impurities or foreign phases.

**Table 1:** The main refinement parameters and detailed crystallographic data of Sr_3_MgSi_2_O_8_ host and Sr_3_MgSi_2_O_8_:0.02Ce^3+^ phosphor

Compound	Sr_3_MgSi_2_O_8_	Sr_3_MgSi_2_O_8_:0.02Ce^3+^
Crystal system	Monoclinic	Monoclinic
Space group	*P*2_1_/*a* (no. 14)	*P*2_1_/*a* (no. 14)
*a* (Å)	13.8724 (9)	13.8779 (69)
*b* (Å)	5.4565 (8)	5.4569 (27)
*c* (Å)	9.4516 (14)	9.4520 (49)
*β* (°)	90	90
2*θ*-interval (°)	5–90	5–90
*R*_wp_ (%)	8.821	8.896
*R*_exp_ (%)	6.871	6.761
*R*_B_	2.154	1.540
GOF	1.284	1.316

**Fig. 3:**
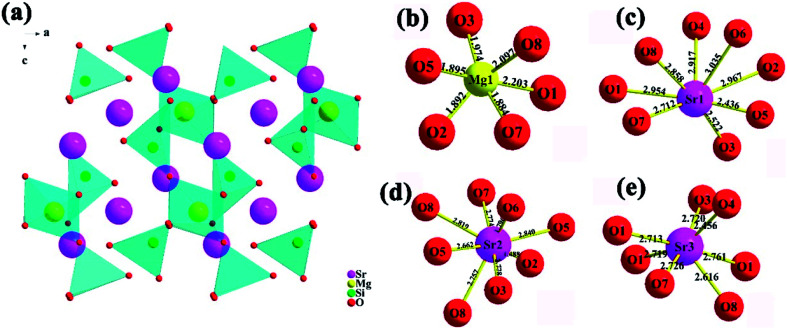
(a) Schematic crystal structure of Sr_3_MgSi_2_O_8_ host; (b–e) coordination environment of Mg^2+^ and Sr^2+^ sites in Sr_3_MgSi_2_O_8_ host.

Based on the refinement results, the crystal structure of Sr_3_MgSi_2_O_8_ host as well as the coordination environment diagram of cation in the host are presented in Fig. 3. The end-to-end [MgO_6_] octahedral and [SiO_4_] tetrahedron make up the main frame of Sr_3_MgSi_2_O_8_ host, while Sr^2+^ ions are embedded between these polyhedron. Because of radius similarity between substitution Ce^3+^ and Mg^2+^/Sr^2+^, Ce^3+^ ions are considered to occupy all the Mg^2+^ and Sr^2+^ sites. To show the local crystal field environment of Ce^3+^ ions, the coordination environment diagram of Mg^2+^ and Sr^2+^ is given in Fig. 3(b–e). It's seen that Mg^2+^ ions occupy six-coordinated site while Sr^2+^ ions occupy three different sites with eight and seven-coordinated environment.

**Fig. 4:**
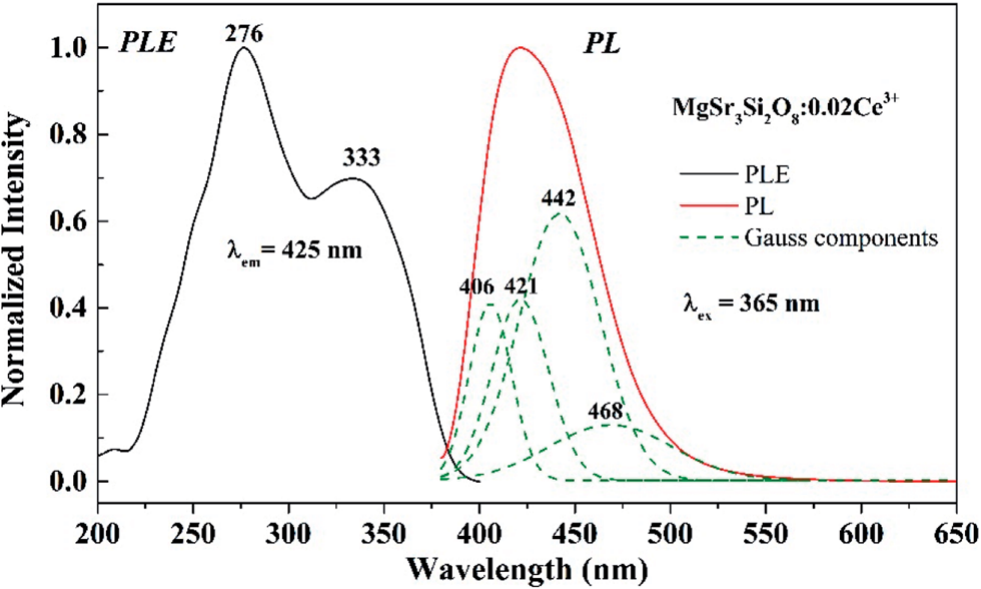
PLE (left) and PL (right) spectra of the Sr_3_MgSi_2_O_8_:0.02Ce^3+^ phosphor; fitting of the emission spectra of the Sr_3_MgSi_2_O_8_:0.02Ce^3+^ phosphor is also outlined by the green dotted line.

The PLE and PL spectra of the Sr_3_MgSi_2_O_8_:0.02Ce^3+^ phosphors are shown in Fig. 4. The PLE spectra of Sr_3_MgSi_2_O_8_:0.02Ce^3+^ monitored at 425 nm exhibited two distinct excitation peaks at 276 and 333 nm, which are assigned to the 4f → 5d transitions of the Ce^3+^ ions. Upon excitation by near UV (n-UV) light (*λ*_ex_ = 365 nm), the PL spectra exhibited an asymmetric blue emission band ranging from 380 to 550 nm with peak center at 425 nm. As shown in Fig. 4, the emission band of Ce^3+^ could be decomposed into four well-separated Gaussian components peaking at 406, 421, 442 and 468 nm, corresponding to the energy 24 649, 23 730, 22 619, 21 336 cm^−1^, respectively. The four Gaussian components may be assigned to the emission from two different Ce^3+^ occupation sites. The energy differences between two peaks are calculated to be 2030 and 2394 cm^−1^, respectively. The energy differences between two components are consistent with the theoretical energy difference between the separate ^2^F_5/2_ and ^2^F_7/2_ levels of the 4f ground state of Ce^3+^ due to shielding by the outer 5s and 5p electrons, which usually present the value as approximately 2000 cm^−1^.^8^

**Fig. 5:**
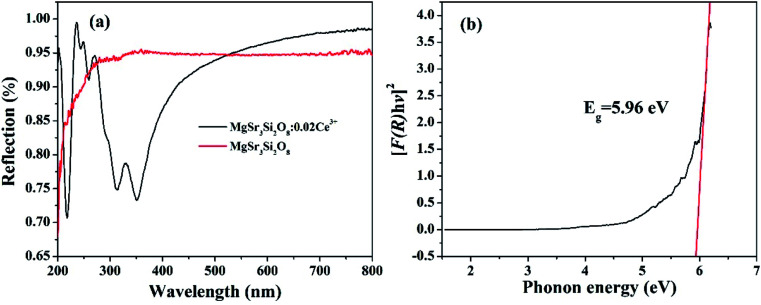
(a) Diffuse reflectance spectra (DRS) of Sr_3_MgSi_2_O_8_ host and Sr_3_MgSi_2_O_8_:0.02Ce^3+^ phosphor; (b) plot of transforming Kubelka–Munk function *versus* the energy of the light adsorbed.

The diffuse reflectance spectra (DRS) of the Sr_3_MgSi_2_O_8_ host as well as the Sr_3_MgSi_2_O_8_:0.02Ce^3+^ phosphor are presented in Fig. 5. The Sr_3_MgSi_2_O_8_:0.02Ce^3+^ phosphor presents several different absorption bands. The absorption band at approximately 220 nm is assigned to the host absorption. The several bands lying at approximately 240, 260, 310 and 350 nm are attributed to the transition from the ground state to different field-splitting 5d levels of Ce^3+^ ions. The peaks at 310 and 350 nm are in agreement with two absorption peaks at 276 and 333 nm in PLE spectra. The absorption peaks corresponding 240 and 260 nm in PLE are not as obvious as those in DRS probably because the two peaks are so weak that they are covered by the strong peak at 276 nm. The band gap of Sr_3_MgSi_2_O_8_ matrix was calculated according to the following equation:^4^

1 [*F*(*R*_∞_*hv*)]^2^ = *C*(*hv* − *E*_g_),

where *hv* indicates the energy per photon, *C* is a proportional constant, and *E*_g_ represents the band gap. As illustrated in Fig. 5(b), the band gap energy of the Sr_3_MgSi_2_O_8_ matrix was estimated to be approximately 5.96 eV.

**Fig. 6:**
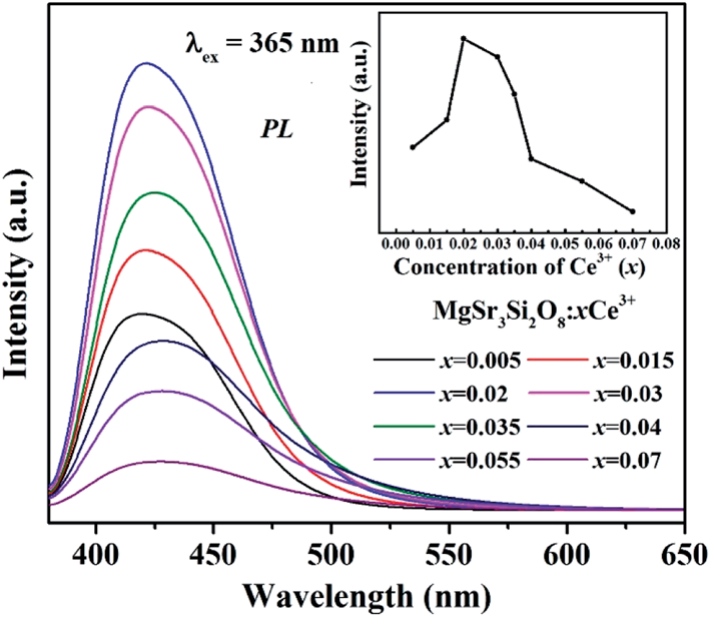
PL spectra of Sr_3_MgSi_2_O_8_:*x*Ce^3+^ phosphors (*x* = 0.005, 0.015, 0.02, 0.03, 0.035, 0.04, 0.055 and 0.07).

The PL spectra of Sr_3_MgSi_2_O_8_:*x*Ce^3+^ phosphors (*x* = 0.005, 0.015, 0.02, 0.03, 0.035, 0.04, 0.055 and 0.07) under 365 nm excitation are presented in Fig. 6. The emission spectra exhibit a single broad band peaks at around 425 nm based on the allowed 4f^6^5d → 4f^7^ transition of Ce^3+^ ions. As shown in the inset in Fig. 6, the emission intensity firstly rises to a maximum then falls with the increase of Ce^3+^ concentration, which is caused by the concentration quenching effect. When the doping concentration of Ce^3+^ increases, the interatomic distance between two Ce^3+^ ions becomes shorter and the energy transfer possibility is enhanced. As a result, the non-radiative transition happens between sensitizers or between sensitizer and activator, which decreases the efficiency and luminous intensity, referred to as the concentration quenching.^20^ The optimum Ce^3+^ concentration is 2 mol%, which indicates that the Sr_3_MgSi_2_O_8_:*x*Ce^3+^ phosphors with optimal efficiency can be obtained at a relatively low quenching concentration.

In terms of the energy transfer between two luminous centers, the transfer mechanism may take place through three modes, including radiation reabsorption, exchange interaction and electric multipolar interaction.^21^ The mechanism of radiation reabsorption is only effective when the fluorescence and absorption spectra are broadly overlapping. Therefore, radiation reabsorption does not occur in this case. It is necessary to obtain the critical distance (*R*_c_) for energy transfer among Ce^3+^ ions to verify the process of energy transfer in this case. According to Dexter,^22^ the value of the critical distance (*R*_c_) can be reckoned *via* the following equation:2
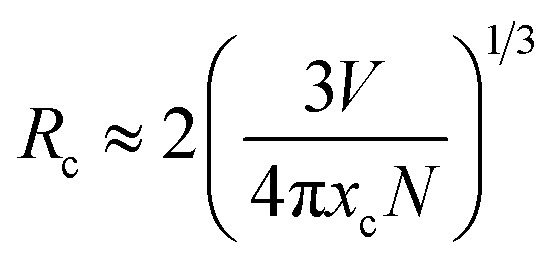
here *V* represents the unit cell volume, *x*_c_ is critical quenching concentration and *N* is the number of the Mg^2+^/Sr^2+^ ions in per unit cell. Critical distance *R*_c_ is calculated as 12.62 Å by adopting the values of *V* = 715.44 Å, *N* = 34 and *x*_c_ = 0.02. Critical distance for the exchange interaction mode is approximately 5 Å. Therefore, the exchange interaction may not play a leading role in the energy transfer within the Sr_3_MgSi_2_O_8_:Ce^3+^ phosphors. Thus, the electric multipolar interactions are dominant in the energy transfer process. According Van Uitert,^23^ the mechanism of the interaction can be explained by the following equation:

3 *I*/*I*_0_ = [1 + *β*(*C*/C*)^*θ*/3^]^−1^

where *I*_0_ represents emission per luminescent center obtained under dilute conditions; *I* represents emission per luminescent center at different rare earth concentration; *C* is the concentration of quenching ions, C* is the critical transfer concentration of quenching ions and *θ* = 6, 8 and 10 corresponds to dipole–dipole (d–d), dipole–quadrupole (d–q), and quadrupole–quadrupole (q–q) interactions, respectively.

From Fig. 6, we can see that emission bands widen with increasing Ce^3+^ concentration, which indicates that more sites luminescence may be involved when Ce^3+^ concentration reach a certain level. Due to different luminescence efficiency at different site, the emission intensity can be also changed. Thus, it can be erroneous to explain the mechanism of the interaction by the eqn (3) easily. The full width at half maximum (FWHMs) of emission spectra are calculated and given in Table 2. It's clear that the FWHMs of emission spectra increase with *x* values, indicating that more sites luminescence may be involved with increasing Ce^3+^ concentration.

**Table 2:** FWHMs of emission spectra of Sr_3_MgSi_2_O_8_:*x*Ce^3+^

*x*	0.005	0.015	0.02	0.03	0.035	0.04	0.055	0.07
FWHM (nm)	66.6	67.2	66.4	67.4	69.6	78.2	79.8	78.4

**Fig. 7:**
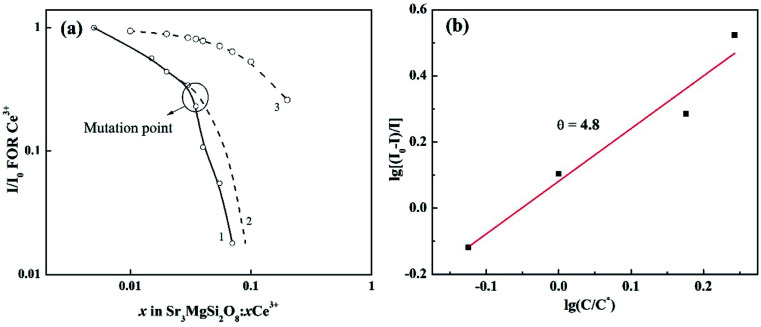
(a) Normalized emission per Ce^3+^ in Sr_3_MgSi_2_O_8_ host *vs.* Ce^3+^ concentration; curve 1 represents the actual case, curve 2 represents the expected case and curve 3 is the relation (1 − *x*)^6^; (b) the relationship between the log(*C*/C*) and log[(*I*_0_ − *I*)/*I*] of Sr_3_MgSi_2_O_8_:*x*Ce^3+^ phosphors.

To have a better understand on the effects of more involved sites on the expression of concentration quenching by eqn (3), the normalized emission intensity *I*/*I*_0_ for per Ce^3+^*vs.* Ce^3+^ concentration is shown in Fig. 7(a). The exchange interaction may not play an important role in the energy transfer because curve 1 doesn't follow the relation (1 − *x*)^6^ (curve 3).^23^ The *I*/*I*_0_ usually shows a continuous smooth reducing trend with quenching ion concentration.^23^ However, the curve 1 suddenly decays with an unexpected rate when *x* reaches 0.035. The mutation point is consistent with that in Table 2, where the FWHMs show an obvious increase after *x* reach 0.035. Hence, it's reasonable to conclude that when *x* reach a certain high level (about 0.035), Ce^3+^ ions may occupy some new sites, in which the Ce^3+^ ions may have lower luminescent efficiency. Hence, the curve 1 decays with a faster rate compared to the one it should be (as shown in curve 2). Due to the interference of this effect, only data at lower *x* values are adopted to calculate the multipolar interactions with eqn (3). The dependence of log[(*I*_0_ − *I*)/*I*] on log(*C*/C*) is plotted in Fig. 7(b). The value of *θ* is finally determined to be 4.8, indicating that the mechanism of energy transfer in Sr_3_MgSi_2_O_8_:*x*Ce^3+^ phosphors is mainly predominated by dipole–dipole interaction.

**Fig. 8:**
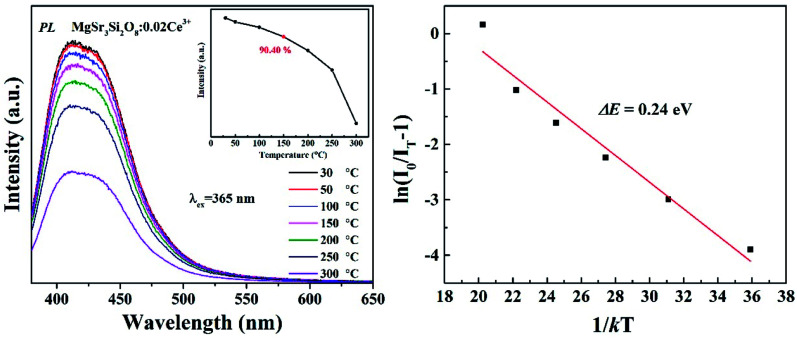
(Left) Temperature-dependent PL spectra of Sr_3_MgSi_2_O_8_:0.02Ce^3+^ phosphor. The inset shows dependence of the emission intensities on temperatures; (Right) the fitting plot of ln(*I*_0_/*I*_*T*_^−1^) and 1/*kT* according to Arrhenius equation.

When functioning, the stability and reliability of a w-LED is finally determined by thermal stability of a phosphor. Good thermal stability of phosphors is always attributed to the rigid three-dimensional structure formed by polyhedron in related literatures. The temperature-dependent luminescent properties of Sr_3_MgSi_2_O_8_:0.02Ce^3+^ phosphors were measured. The activation energy, which refers to the energy barrier for non-radiative transition is an important value to estimate the thermal stability of a phosphor. Hence, the activation energy was calculated using the Arrhenius equation:5
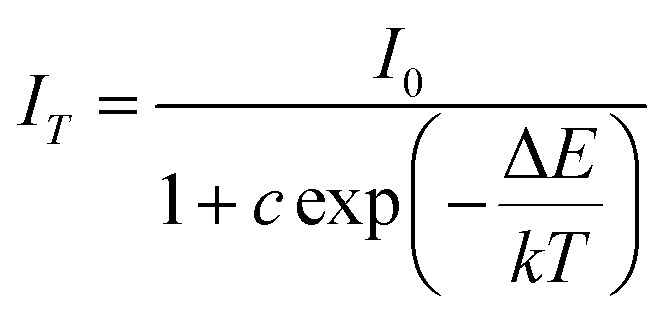
where *I*_0_ and *I*_*T*_ are the emission intensity at room and measurement temperature *T*, respectively, *c* is a constant, *k* is the Boltzmann constant (8.617 × 10^−5^ eV K^−1^) and Δ*E* is the activation energy for the thermal quenching. The temperature-dependent spectra and calculated activation energy (Δ*E*) are shown in Fig. 8. For Sr_3_MgSi_2_O_8_:0.02Ce^3+^, Δ*E* was determined to be 0.24 eV, and the luminous intensity at 150 °C was 90.4% of that at room temperature. It's obvious that the Sr_3_MgSi_2_O_8_:0.02Ce^3+^ phosphor has an excellent thermal stability.

**Fig. 9:**
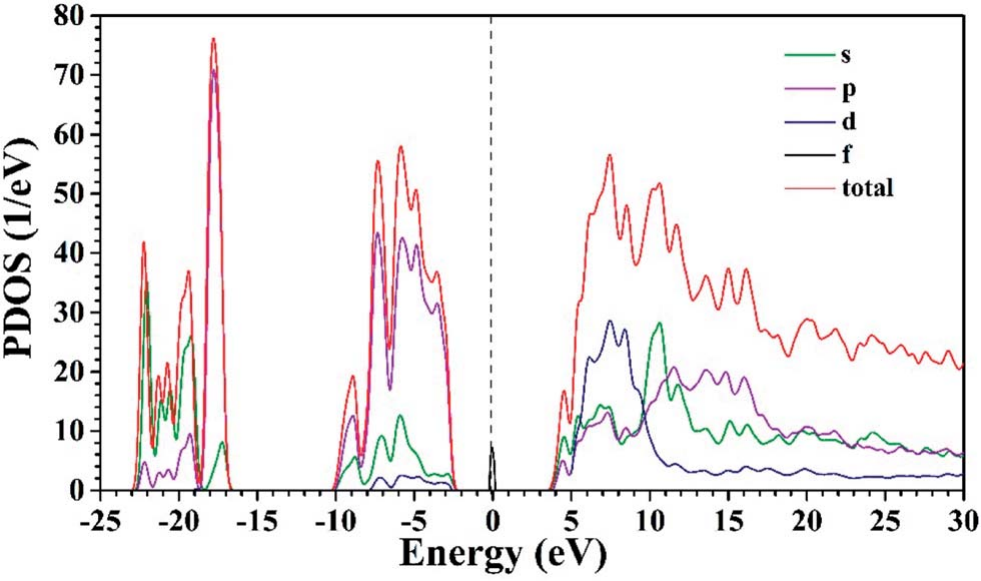
Projected electronic density of states (PDOSs) of Sr_3_MgSi_2_O_8_:0.02Ce^3+^.

So far, several thermal quenching processes have been proposed including thermal relaxation through the crossing point based on the configurational coordinate model, thermal ionization from the emitting 5d^1^ levels to the conduction band and direct electron transfer from (higher) 5d levels to the conduction band with no activation energy (photoionization of the Ce^3+^ ion) prior to relaxation to the lowest 5d^1^ state.^8,24^ Although the configuration diagram is one of the most adopted model, some papers make an argument that the photoionization in the real mechanism for thermal quenching.^25,26^ Considering this matter, the energy level position of Ce^3+^ ion in Sr_3_MgSi_2_O_8_ host is calculated by first principle method. Fig. 9 illustrates the projected density of states (PDOSs) of Sr_3_MgSi_2_O_8_:0.02Ce^3+^ phosphor. The *E*_g_ is 5.72 eV. This agrees with 5.96 eV which is got from DRS. The energy gap between the 4f level of Ce^3+^ and the top of valence band was calculated to be 2 eV.

**Fig. 10:**
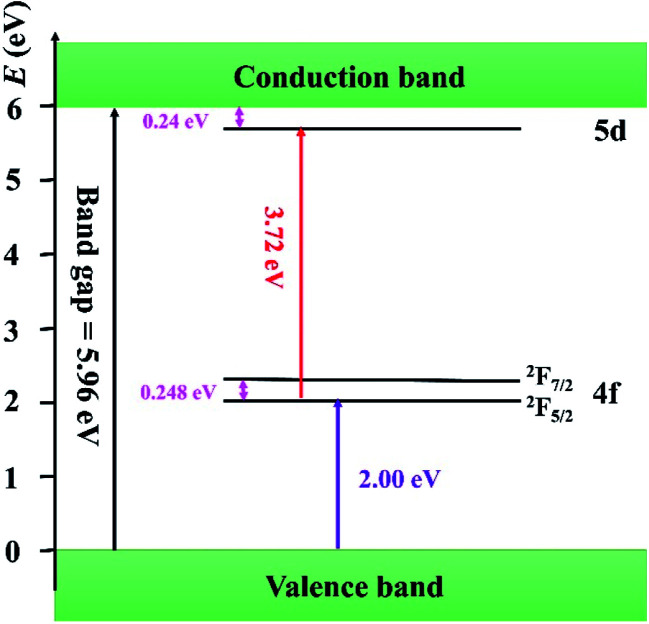
Schematic energy diagram of Ce^3+^ ion in Sr_3_MgSi_2_O_8_ host.

The diagram of the energy level position of Ce^3+^ ion in Sr_3_MgSi_2_O_8_ host is given in Fig. 10. The energy gap between 4f of Ce^3+^ and the top of the valence band (2.00 eV) is obtained from PDOSs. The excitation energy (3.72 eV) is obtained from PLE spectra. The typically energy difference of ^2^F_5/2_ and ^2^F_7/2_ is around 2000 cm^−1^ (0.248 eV), which is also considered in the scheme. Considering these energy levels, it's reasonable to suppose that thermal ionization from the emitting lowest 5d levels to the conduction band is the real mechanism of concentration quenching in the Sr_3_MgSi_2_O_8_ because the small energy gap between un-relaxed lowest 5d levels and the conduction band (0.24 eV) may not result in such a high quenching temperature.

**Fig. 11:**
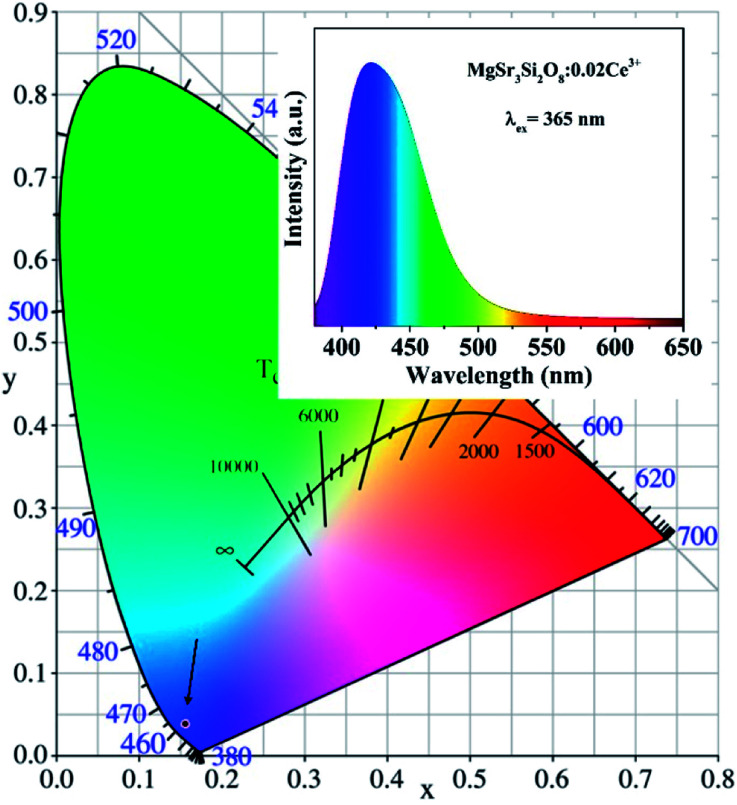
CIE chromaticity diagrams of Sr_3_MgSi_2_O_8_:0.02Ce^3+^ phosphor; the inset shows the PL spectra of Sr_3_MgSi_2_O_8_:0.02Ce^3+^ phosphor with wavelength-dependent colors.

To show the real color of the as-prepared phosphors, the color rendering index (CIE) chromaticity coordinates of the Sr_3_MgSi_2_O_8_:0.02Ce^3+^ phosphor was calculated and shown in Fig. 11. It can be seen that the CIE coordinate of Sr_3_MgSi_2_O_8_:0.02Ce^3+^ phosphor lie in the blue region (0.1561, 0.0389) which demonstrates that the Sr_3_MgSi_2_O_8_:0.02Ce^3+^ can serve as a blue phosphor for w-LEDs. The PL spectra of Sr_3_MgSi_2_O_8_:0.02Ce^3+^ phosphor with wavelength-dependent colors is also presented in the inset.

To evaluate the practicability of title phosphor, the quantum efficiency (QE) of the optimal sample Sr_3_MgSi_2_O_8_:0.02Ce^3+^ is measured to be 85%, which can be made even higher by improving synthesis conditions.^27^ As Fig. 5(a) shows, Ce^3+^ doped Sr_3_MgSi_2_O_8_ phosphor has strong absorption in the UV region, which matches UV chip very well. In general, with excellent thermal stability, high QE and strong absorption in UV region, the title phosphor is deserved to be a potential blue phosphor for UV chip based w-LED.^28,29^

## 4. Conclusion

The Ce^3+^ doped phosphor can be obtained by traditional high-temperature solid-state reaction method. The title phosphor crystallize in a monoclinic crystal system. The mechanism of concentration quenching was discussed and the energy transfer between Ce^3+^ ions was determined to be quadrupole–quadrupole interactions. The Sr_3_MgSi_2_O_8_:0.02Ce^3+^ title phosphor presents an excellent thermal stability. When temperature rises to 150 °C, the emission intensity can still remain 90% of that in room temperature. The thermal quenching mechanism was considered to be thermal ionization and a schematic energy levels position of Ce^3+^ ion in Sr_3_MgSi_2_O_8_ host is drowned to illuminate the thermal ionization process in title phosphor.

## Conflicts of interest

There are no conflicts to declare.

## Supplementary Material
